# Shell Condition and Survival of Puget Sound Pteropods Are Impaired by Ocean Acidification Conditions

**DOI:** 10.1371/journal.pone.0105884

**Published:** 2014-08-27

**Authors:** D. Shallin Busch, Michael Maher, Patricia Thibodeau, Paul McElhany

**Affiliations:** Conservation Biology Division, Northwest Fisheries Science Center, National Marine Fisheries Service, National Oceanic and Atmospheric Administration, Seattle, Washington, United States of America; University of California Santa Barbara, United States of America

## Abstract

We tested whether the thecosome pteropod *Limacina helicina* from Puget Sound, an urbanized estuary in the northwest continental US, experiences shell dissolution and altered mortality rates when exposed to the high CO_2_, low aragonite saturation state (Ω_a_) conditions that occur in Puget Sound and the northeast Pacific Ocean. Five, week-long experiments were conducted in which we incubated pteropods collected from Puget Sound in four carbon chemistry conditions: current summer surface (∼460–500 µatm CO_2_, Ω_a_≈1.59), current deep water or surface conditions during upwelling (∼760 and ∼1600–1700 µatm CO_2_, Ω_a_≈1.17 and 0.56), and future deep water or surface conditions during upwelling (∼2800–3400 µatm CO_2_, Ω_a_≈0.28). We measured shell condition using a scoring regime of five shell characteristics that capture different aspects of shell dissolution. We characterized carbon chemistry conditions in statistical analyses with Ω_a_, and conducted analyses considering Ω_a_ both as a continuous dataset and as discrete treatments. Shell dissolution increased linearly as aragonite saturation state decreased. Discrete treatment comparisons indicate that shell dissolution was greater in undersaturated treatments compared to oversaturated treatments. Survival increased linearly with aragonite saturation state, though discrete treatment comparisons indicated that survival was similar in all but the lowest saturation state treatment. These results indicate that, under starvation conditions, pteropod survival may not be greatly affected by current and expected near-future aragonite saturation state in the NE Pacific, but shell dissolution may. Given that subsurface waters in Puget Sound’s main basin are undersaturated with respect to aragonite in the winter and can be undersaturated in the summer, the condition and persistence of the species in this estuary warrants further study.

## Introduction

Global oceans have absorbed about a third of the anthropogenically released carbon dioxide [1], [2], [3]. The accumulation of carbon dioxide in seawater has lowered average global ocean pH from 8.2 to 8.1 and decreased the concentration of carbonate ions [1]. Projections of carbon dioxide emissions indicate that by year 2100, surface ocean pH will be 0.3–0.4 units lower than today, and carbonate ion concentration will decrease by about 50% [4], [5], [6], [7]. Changes in the carbonate ion concentration of seawater can affect how readily calcium carbonate structures accrete or dissolve, a property reflected in seawater saturation state, Ω. Once the carbonate ion concentration is low enough (Ω<1), dissolution of calcium carbonate structures is favored.

Ocean acidification can affect a variety of physiological processes in marine organisms, from photosynthesis to neuronal signaling [8], [9]. Although there is much variation in response to ocean acidification among marine species, in general, laboratory studies indicate that ocean acidification has negative effects on survival, calcification, growth, and reproduction; these effects are larger for calcifying species than for non-calcifiers [10]. Differential response of marine species to ocean acidification has the potential to change the structure of marine communities, as has been observed in natural experiments, modeling exercises, and Earth’s history [11], [12], [13], [14].

Pteropods are small, holoplanktonic marine snails with aragonitic calcium carbonate shells, and are important prey species in many marine food webs [15], [16], [17], [18]. All available research indicates that growth rate and calcification of shelled pteropods decline with ocean acidification [19], [20], [21], [22], [23]. Pteropods can build shell when seawater is undersaturated with respect to aragonite, but their shells also experience dissolution [20], [21], [22], [23], [24], [25], [26]. Pteropod mortality may increase as pCO_2_ increases, but studies done to date, only in high-latitude regions, have yielded uncertain and contradictory results [22], [23], [24]. Over the past few decades, the abundance of subarctic pteropods (*Limacina* spp.) in the North Pacific off of Canada’s Vancouver Island has declined in 3 of 4 locations, while abundance of subtropical planktivorous pteropods (*Clio* spp.) and predatory pteropods (*Clione* spp.) has increased offshore of the southern part of Vancouver Island and remained unchanged elsewhere in the region [27].

*L. helicina* is distributed throughout polar and some subpolars seas, and is the most abundant pteropod species in marine waters of the US Pacific Northwest. *L. helicina* uses a mucus web to gather food, and, in Arctic waters, the species is an omnivore in autumn and winter and an herbivore in spring and summer [28]. In North Pacific waters, pteropods are prey for fishes such as anchovy (*Engraulis mordax)*, herring (*Clupea pallasii)*, jack mackerel (*Trachurus symmetricus*), sablefish (*Anoplopoma fimbria*), and pink, coho, chum, and sockeye salmon (*Oncorhynchus gorbuscha*, *O. kisutch*, *O. keta*, *O. nerka*) [15], [16], [17], [18]. Pteropods are also eaten by other zooplankton, squid, whales, and birds. Many pteropod populations undergo diel vertical migration, feeding in surface waters at night and retreating to deeper waters during the day to avoid predation. The diel migration for *L. helicina* is shallower in the Pacific Northwest region (100 m) [27] than other parts of the species’ range (150–250 m) [29]. *L. helicina* forms a delicate aragonitic shell with a periostracum covering [30], and the species is globally a major contributor of inorganic and organic carbon fluxes to the deep ocean [31], [32]. In Arctic waters, it has a 1-yr life-cycle [28]. Natural history information on North Pacific *L. helicina* is scant.

Due to natural oceanographic processes, aragonite saturation horizons in North Pacific waters are some of the shallowest in the world [5], [33], [34], [35]. Changes in ocean circulation in the California Current have compounded recent declines in aragonite saturation state [36]. Large-scale, coastal upwelling occurs each spring and summer in the California Current System. Upwelled waters are naturally high in both nutrients and CO_2_, and ocean acidification adds to the CO_2_ load of these waters [34]. Global climate change may increase upwelling favorable winds in this region [37]. Upwelling has intensified in the southern California Current System in recent decades [38]. Recent observations of the California Current found that ocean acidification has changed ocean chemistry enough that waters undersaturated with respect to aragonite now reach the surface in some locations when upwelling occurs [34]. Without ocean acidification, undersaturated waters would be 50 m deeper than they are currently [34]. Models project that by 2050, large sections of nearshore (0–10 km from coast) water from 0–60 m will be undersaturated with respect to aragonite during the entire summer upwelling season and more than half of the nearshore water mass will be undersaturated throughout the year [39]. Thus, upper, nearshore waters of the California Current System are likely to develop undersaturated conditions at a similar timeframe to the Southern Ocean and potentially faster than surface Arctic waters [39].

Low pH water from the California Current feeds into Puget Sound, a fjordal estuary in the northwestern continental US. Once in Puget Sound, ocean carbon chemistry is influenced by low-pH, low-alkalinity freshwater inputs; nutrient run-off caused by agriculture and urban development and the influence of these nutrients on biological activity; and local oceanographic processes that lead to stratification and restrictions in flow [40]. Because of these inputs and processes, Puget Sound waters have low pH and aragonite saturation states. Since the Industrial Revolution, ocean acidification in Puget Sound has caused a decrease in pH of 0.05–0.15 units and surface aragonite saturation state of 0.09–0.33 [40]. In Puget Sound’s main basin in the summer, surface waters (depth<8 m) are supersaturated with respect to aragonite (Ω_a_ = 1.01–2.79), but waters below 50 m range between under- and supersaturated (Ω_a_ = 0.86–1.35) [40]. In the winter, the entire water column in the main basin is undersaturated with respect to aragonite (Ω_a_ = 0.79–0.95) [40].

We test whether the vertically migrating, thecosome pteropod *Limacina helicina* collected in Puget Sound is sensitive to levels of pCO_2_ found currently in the estuary and expected more frequently in the future [41]. Such conditions are also representative of current and future upwelling events in the North Pacific. This is the first study to test the sensitivity of temperate North Pacific *L. helicina* to different current pCO_2_ levels and those expected with ocean acidification. By evaluating their sensitivity to carbon chemistry conditions, it is also a first step towards understanding how North Pacific pteropods persist in the corrosive conditions that typify the northern California Current. Adaptation to different environmental conditions has been documented previously between populations of pteropods [42], and differential sensitivity to ocean acidification has also been found within other invertebrate species and among pteropod species [43], [44], [45]. We test the hypothesis that shell condition is linked directly to aragonite saturation state, and expect dissolution only in the treatments where Ω_a_<1. Due to uncertainties in the relationship between survival and carbon chemistry conditions from prior research [22], [23], [24], we expect that *L. helicina* survival will not be affected by carbon chemistry treatment in laboratory conditions.

## Materials and Methods

Pteropods used in this study were collected from Puget Sound using collection permits from the Washington Department of Fish and Wildlife. No specific requirements for planktonic molluscs are included in national animal care guidelines.

We conducted five experiments on *L. helicina* in the summer of 2012, two in May and three in July. We collected adult pteropods (mean shell diameter = 1.94 mm, standard deviation = 0.25 mm, N = 68 individuals) from Port Susan in Puget Sound, Washington (48.1° N, 122.4° W), using 1 m-diameter ring nets with mesh sizes of 335, 580, or 2000 µm towed obliquely at 2–3 knots between 5–40 m depth. Collecting trips occurred from twilight to early night. Pteropods were transported to the NOAA Northwest Fisheries Science Center in a cooler filled with aerated seawater. For the May experiments, pteropods were kept in the cooler overnight. For the July experiments, pteropods were placed into 4.5-L PET plastic jars immediately upon arrival in the lab, and those jars were connected to a recirculating seawater system with ∼460–500 µatm CO_2_. The morning following collection, we haphazardly distributed swimming or otherwise active pteropods into 4.5-L PET plastic jars at densities of 33–40 pteropods per jar, depending on the experiment (Table 1). This density is higher than reported *L. helicina* density in the wild [27], [46], [47], but lower than the maximum density reported for the congener *L. retroversa* [48]. Compared to other studies that explore the impact of ocean acidification on pteropod survival, density was similar to that used in one study [24] and 2.5 to 20 times less than the two others [22], [23]. Each jar acted as a replicate, and replicates were spread equally across all treatments in a given experiment (Table 1). We gradually increased dissolved CO_2_ from ∼460–500 µatm to treatment conditions in 2 h, which resembles the rapid drop in pH and aragonite saturation state that coastal organisms experience when they vertically migrate downward or upwelled waters invade the upper ocean [35]. We did not feed the pteropods due to the technical difficulties of developing proper feeding conditions for pteropods in laboratory settings, especially in jars with water flow.

**Table 1:** Treatment conditions during experiments.

Exp.	Dates	N_replicates_	N_jar_	N_shells_	Salinity	Temp.	System pH		Jar pH	pCO_2_ *	Ω_a_ *	TA	DIC
					(psu)	(°C)	Durafet	Spec	Spec	(µatm)		(µmol/kg)	(µmol/kg)
1	5/10–5/16	3	40	35	31.5	12.12±0.15	8.01±0.02	7.97	–	491	1.7	2239.6	2092.9
				42	31.5	12.13±0.18	7.49±0.01	7.50	–	1576	0.62	2235.3	2236.5
2	5/17–5/23	4	40	23	31.8	12.04±0.07	7.98±0.05	7.95±0.03	7.92	502±34	1.64±0.10	2237.8	2076.3
				33	31.8	12.04±0.06	7.76±0.00	7.79±0.02	7.81	766±40	1.17±0.06	2239.2	2140.2
				23	31.8	12.05±0.07	7.48±0.01	7.46±0.03	–	1725±122	0.58±0.04	2230.5	2246.7
3	6/29–7/5	4	33	46	31.9	12.08±0.08	8.02±0.00	7.97	7.99	462	1.50	2092.1	1940.5
				26	31.9	12.07±0.08	7.47±0.01	7.46	7.46	1614	0.50	2085.6	2080.5
				4	31.9	12.09±0.09	7.19±0.01	7.22	7.21	2815	0.30	2084.5	2147.0
4	7/3–7/9	2	39	13	29.6	12.09±0.09	8.02±0.00	7.98±0.00	7.97	460±3	1.51±0.01	2101.5	1943.6
				14	29.6	12.08±0.07	7.47±0.00	7.47±0.02	7.47	1579±50	0.52±0.02	2097.4	2093.4
				0**	29.6	12.11±0.12	7.19±0.01	7.19±0.05	7.12	3057±342	0.28±0.03	2092.2	2165.3
5	7/6–7/12	3	40	24	29.7	12.12±0.13	8.02±0.00	7.97±0.00	7.96	460±4	1.53±0.01	2114.3	1956.6
				21	29.7	12.09±0.09	7.47±0.00	7.47±0.02	7.46	1585±59	0.53±0.01	2108.1	2100.7
				7	29.7	12.12±0.12	7.18±0.01	7.14±0.02	7.15	3421±174	0.25±0.01	2101.7	2211.1

Exp. stands for experiment, temp. for temperature, spec. for spectrophotometer, TA for total alkalinity, and DIC for dissolved inorganic carbon. N_replicates_ is the number of replicate jars in each treatment, N_jar_ is the number of pteropods per jar, and N_shells_ is the number of shells in a treatment scored for shell condition.

* Ω_a_ and pCO_2_ values calculated from DIC and pH measured via spectrophotometry.

** No pteropods survived this treatment in experiment 4.

We chose the experimental treatments based on pCO_2_ conditions observed in Puget Sound [40] and projected for the future. The ∼460–500 µatm pCO_2_ treatment represents summer surface conditions. Because of the variability in pCO_2_ in Puget Sound, we use two treatments, ∼760 and ∼1600–1700 µatm pCO_2_, to represent either current deep water conditions or surface conditions during upwelling. The ∼2800–3400 µatm pCO_2_ treatment represents future deep water conditions or future surface conditions during upwelling. Future pCO_2_ conditions in Puget Sound were approximated by assuming an addition of dissolved inorganic carbon (DIC) at a concentration of 90 µmol/kg seawater above current levels (P. McElhany and J. Reum, unpublished data).

After seven days of incubation, we terminally sampled each experiment. Live and dead pteropods in each jar were counted and preserved separately in 70% ethanol. Most live individuals were clearly identifiable by movement of the wings. All pteropods without wing movement were visualized under a dissecting microscope to detect movement of the internal organs. We rated the shell condition of pteropods that lived through the seven-day experiment, using the methodology presented in Lischka et al. [22] with one modification: we immersed samples in 6% sodium hypochlorite for 60 hr to sufficiently clean tissue from the shells. The scoring regime presented in Lischka et al. [22] assesses five shell characteristics, with either four or five point scales (scores 0–3 or 0–4): shell transparency/opaqueness, shell transparency/brownness, scarred structures, corrosion, and number of perforations [22]. Higher scores indicate more shell corrosion. We consider overall shell condition to be the sum of the scores for these five characteristics. Selection of shells for scoring was done by two observers who were blind to treatment condition and selected shells haphazardly. All shells selected for scoring were scored by a single observer, who was blind to treatment conditions, using a Nikon SMZ 745T stereoscope. We preserved pteropods from 4 of the 5 collection trips in 70% ethanol on day 0 of the experiments to examine shell state of the free-living population, and rated 17 haphazardly chosen shells from this collection for shell condition.

### Experimental system and carbon chemistry measurements

**Figure 1:**
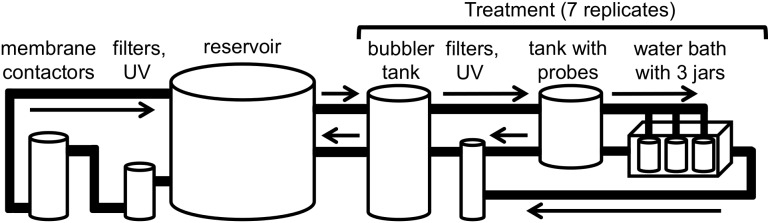
Simplified schematic of ocean acidification experimental system at NOAA’s Northwest Fisheries Science Center.

We conducted these experiments at the NOAA Northwest Fisheries Science Center ocean acidification laboratory (Figure 1). Seawater used in the ∼20,000 L experimental system was collected from Elliot Bay, Seattle, Washington, and the majority of it housed in a large reservoir. Water in the reservoir was filtered to 1 µm, exposed to UV, and degassed using Liqui-Cel membrane contactors (Membrana, Charlotte, North Carolina). A Honeywell conductivity probe monitored seawater salinity in the system. Treatment conditions were controlled by a program built in LabView Software (National Instruments, Austin, Texas) and maintained by bubbling one of four gases: air, CO_2_-free air, CO_2_, and O_2_. CO_2_-free air was generated with CO_2_ adsorbers (Twin Towers Engineering, Broomfield, Colorado). Temperature, pH, and dissolved oxygen in each treatment were continuously monitored with temperature, Durafet pH, and dissolved oxygen transmitter probes, respectively (Honeywell Process Solutions). The flow-through, pteropod-rearing jars (4.5 L, CO_2_-impermeable PET plastic) were connected to the treatment systems, and flow through each jar was approximately 6 L/hr. Jars were immersed in water baths with sufficient flow to maintain the target temperature (12°C) and kept in darkness behind black-out curtains. In each experiment, all jars for a given pCO_2_ treatment were in the same water bath; technical difficulties prevented the interspersing of pCO_2_ treatments among water baths. When we conducted these experiments, the experimental system contained some components that were later found toxic to herring (*Clupea pallasii*), copepod (*Calanus pacificus*), krill (*Euphausia pacifica*) larvae, but not to oyster (*Crassostrea gigas*) and geoduck (*Panopea generosa*) larvae or early life stages of Dungeness (*Metacarcinus magister)* and pygmy rock crab (*Cancer oregonensis).*

We measured three carbon-chemistry parameters (pH, DIC, and total alkalinity) for each study. Durafet pH probes in each experimental treatment continuously recorded pH, and every probe was calibrated at 12°C with a pH-certified Tris buffer (Dickson Laboratory, Scripps Institution of Oceanography). pH conditions in all treatments and rearing jars were verified by taking discrete samples for pH measurement using a spectrophotometer (Ocean Optics USB 2000+ Fiber Optic Spectrometer) and m-cresol purple dye (Sigma Aldrich). Discrete water samples for measurement of total alkalinity and DIC were taken once during each experiment, and these samples were analyzed at NOAA Pacific Marine Environmental Laboratory or the University of Washington’s Friday Harbor Laboratory. Standard operating procedures were followed for all carbon chemistry analyses [49]. We used DIC and spectrophometric pH measurements to estimate pCO_2_ and aragonite saturation state (Ω_a_) with CO2sys version 2.1 [50], using the K1 and K2 constants from Lueker et al. [51], KHSO_4_ constant from Dickson [52], [B]_T_ from Uppstrom [53], and the total pH scale.

### Statistical analysis

We assessed whether survival and shell condition over the 7-day study varied with treatment conditions using mixed-effects logistic regression with the lme4 package (method glmer) in the R statistical program [54]. Ω_a_ was used as the metric to characterize treatments (fixed effect), and, in separate sets of models for each dataset, we considered Ω_a_ as either a continuous variable or as a discrete treatment. Considering Ω_a_ continuously allows evaluation of linear trends in the data, and considering Ω_a_ as discrete treatments enables analyses that can distinguish significant differences among the four carbon chemistry treatments. We included two random effects in the model sets: experiment and jar within experiment. The best-fit models for the discrete and continuous Ω_a_ model sets were selected based on AICc values of alternative models [55]. A logit scale was used for survival data, and a linear scale for the shell condition data. Hosmer-Lemeshow tests were used to assess if survival data (a binomial data set) met the assumptions of the statistical models. A Fligner-Killeen test was used to assess homogeneity of variance of the residuals from the discrete model on the shell condition data set. The sum score of the five shell condition characteristics was used for shell condition statistical analysis.

## Results

The 4 treatments included in these experiments were distinct in pH, DIC, pCO_2_, and Ω_a_ (Table 1). pH values for each treatment were consistent among the 5 experiments and varied little in each treatment over the course of each experiment (standard deviation ≤0.05). Mean pH values from the Durafet sensors were consistent with discrete spectrophotometric measurements of pH from each system and, when available, inside each jar, indicating that the Durafet pH probe measurements were accurate and that carbon chemistry conditions in the rearing jars were consistent with treatment system pH. Alkalinity in all treatments within each experiment was similar, but varied by ∼140 µmol/kg between the experiments in May and July due to use of different batches of seawater. Differences in alkalinity among experiments led to variation in DIC, pCO_2_, and Ω_a_ for each treatment among experiments. Two of the treatments were oversaturated with respect to aragonite and two of the treatments were undersaturated with respect to aragonite. Mean temperature in the treatments ranged from 12.04–12.13°C, with small variation in each treatment over each experiment (standard deviation <0.2°C).

**Figure 2:**
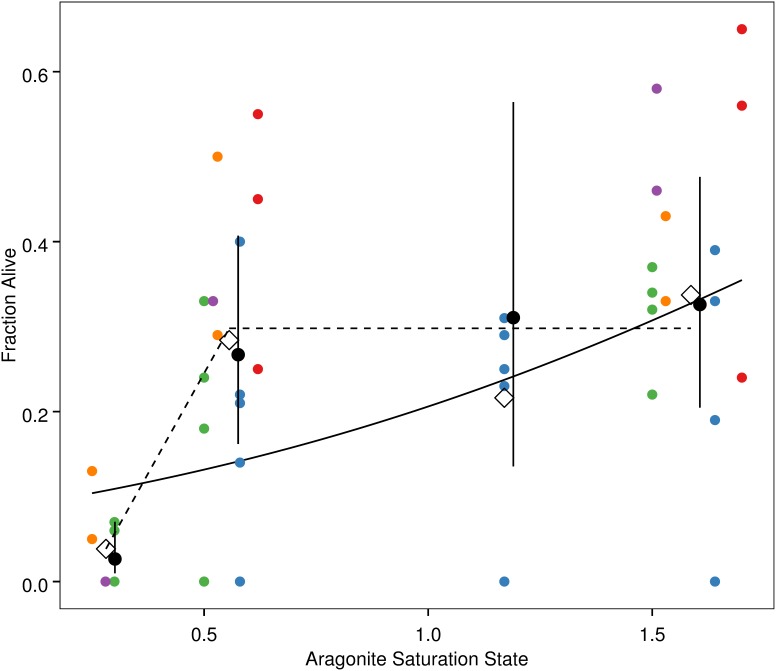
Pteropod survival as a function of Ω_a_. Small colored circles: fraction surviving within an individual jar, with May experiments in warm colors and July experiments in cool colors; open diamonds: overall fraction of pteropods surviving at the mean Ω_a_ for a given treatment; small black circles with vertical bars: expected value and 95% confidence intervals calculated from the best-fit discrete mixed effects model, with small x-axis offset from the treatment mean Ω_a_; solid line: best-fit linear relationship from continuous mixed effects model; dashed line: hypothetical “broken-stick” relationship.

The best-fit model for the continuous Ω_a_ analysis of survival data included Ω_a_ as a fixed effect and jar within experiment as a random effect (Table 2). The slope of this relationship was positive (proportion live in a jar = Ω_a_ * 1.07–2.52) and significantly different from zero (Z = −4.056, p<0.001; Figure 2). For the discrete Ω_a_ analyses of survival data, the best-fit model included Ω_a_ as a fixed effect and experiment and jar within experiment as random effects. Based on comparison of 95% confidence intervals, survival rates in the Ω_a_≈1.59, Ω_a_≈1.17, and Ω_a_≈0.56 treatments were similar and higher than in the Ω_a_≈0.28 treatment. A hypothetical relationship between Ω_a_ and survival based on the discrete analysis is illustrated in Figure 2 as a “broken stick”, which characterizes survival in treatments with Ω_a_≥0.56 using the mean for these treatments and highlights the potentially rapid decrease in survival below this value by drawing a line between mean survival in treatments with Ω_a_≥0.56 to mean survival of the Ω_a_≈0.28 treatment. Results from Hosmer-Lemeshow tests indicate that the survival data are consistent with the assumptions of the models (χ^2^ = 0.67 for the continuous Ω_a_ model, χ^2^ = 0.16 for the discrete Ω_a_ model; number of groups = 10).

**Table 2:** AICc scores for generalized linear mixed effects models of pteropod survival and shell dissolution with aragonite saturation state (Ω_a_) treated as either a continuous or discrete variable.

	AICc score			
	Survival		Shell dissolution	
Model Treatment condition data organization:	Continuous	Discrete	Continuous	Discrete
Response = Ω_a_+(exp) +intercept	1803	1727	1127	1100
Response = Ω_a_+(jar) +intercept	**1687**	1676	**1094**	**1082**
Response = Ω_a_+(exp) +(jar)+intercept	1688	**1674**	1097	1084
Response = Ω_a_+(exp)+(jar) +(exp)×Ω_a_+intercept	1690	1676	1099	1086
Response = Ω_a_+(exp)+(jar) +(jar)×Ω_a_+intercept	1690	1676	1099	1086
Response = Ω_a_+(exp)+(jar) +(jar)×Ω_a_+(exp)×Ω_a_+intercept	1692	1678	1101	1088

The lowest AICc score for each data set (column) is in bold. In the model equations, parentheses indicate random effects. Exp stands for experiment.

**Figure 3:**
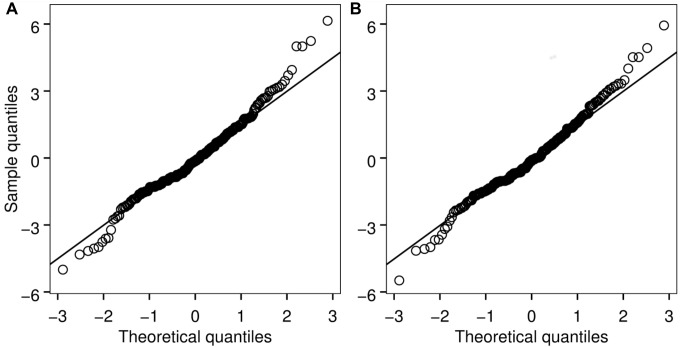
Normal quantile-quantile plot of shell condition model residuals (discrete model, *a*; continuous model, *b*). Solid line shows theoretical conditions in which model residuals are normally distributed. Open circles show actual sample values. Shapiro-Wilks test results indicate that the model residuals deviate from exact normality (p = 0.001 for models).

**Figure 4:**
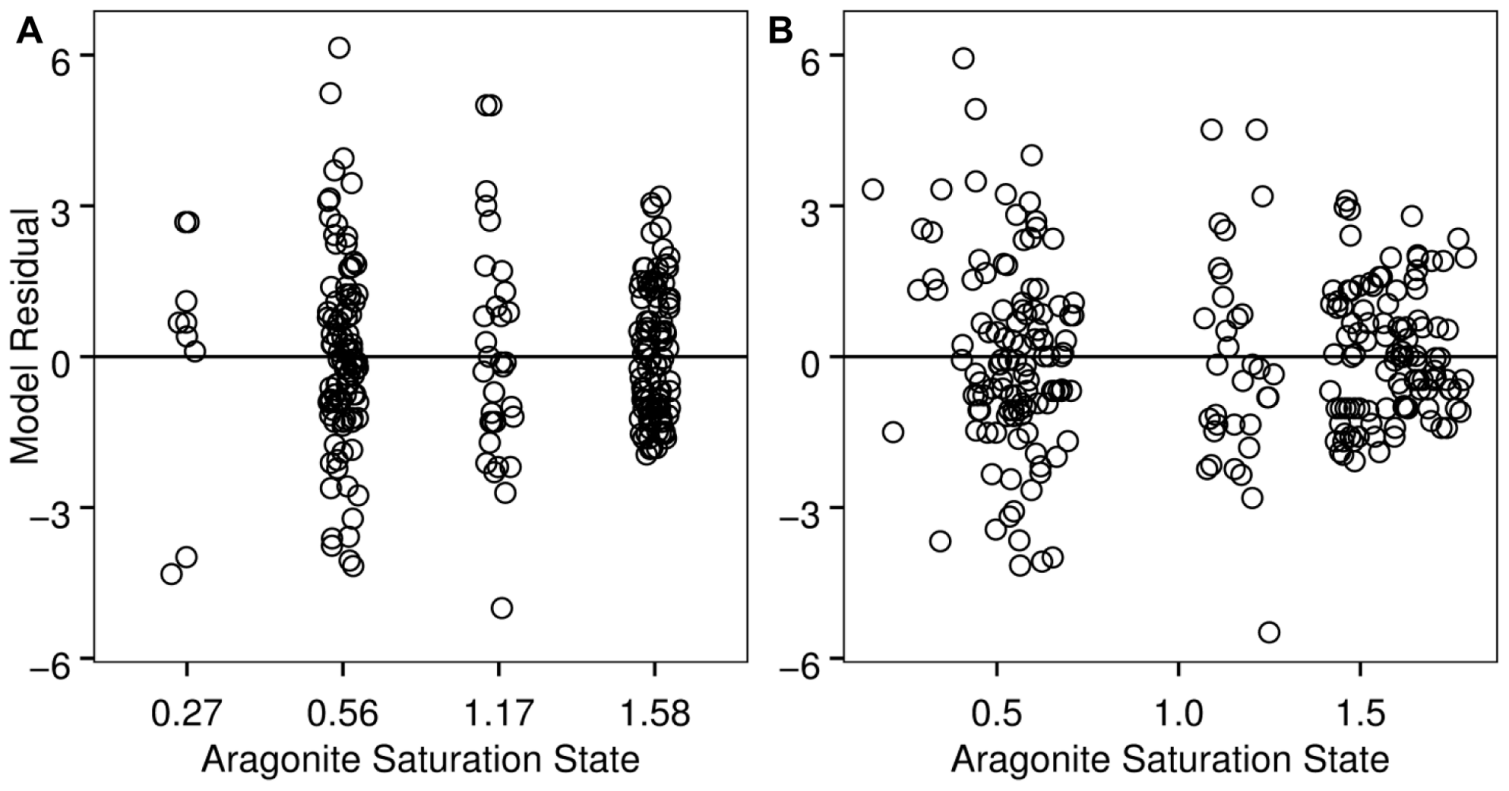
Shell condition model residuals as a function of Ω_a_ (discrete model, *a*; continuous model, *b*). Data are shown with a small x-axis offset from the treatment mean Ω_a_ for better visualization of overlapping points. The plots indicate that the data are reasonably consistent with model assumptions of homoscedasticity.

**Figure 5:**
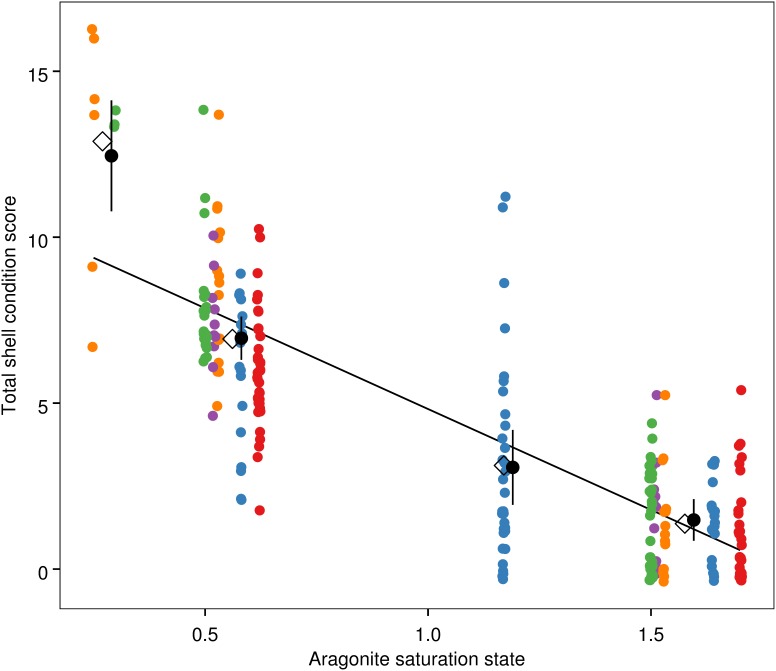
Pteropod sum shell condition score as a function of Ω_a_. Small, colored circles show the sum score for individual pteropods, with May experiments in warm colors and July experiments in cool colors. These points were plotted with small random offsets to prevent overlap. Open diamonds: treatment mean at mean Ω_a_ for a given treatment; small, black circles with vertical bars: expected value and 95% confidence intervals calculated from the best-fit discrete mixed effects model, with a small x-axis offset from the treatment mean Ω_a_; solid line: linear relationship from continuous mixed effects model.

**Figure 6:**
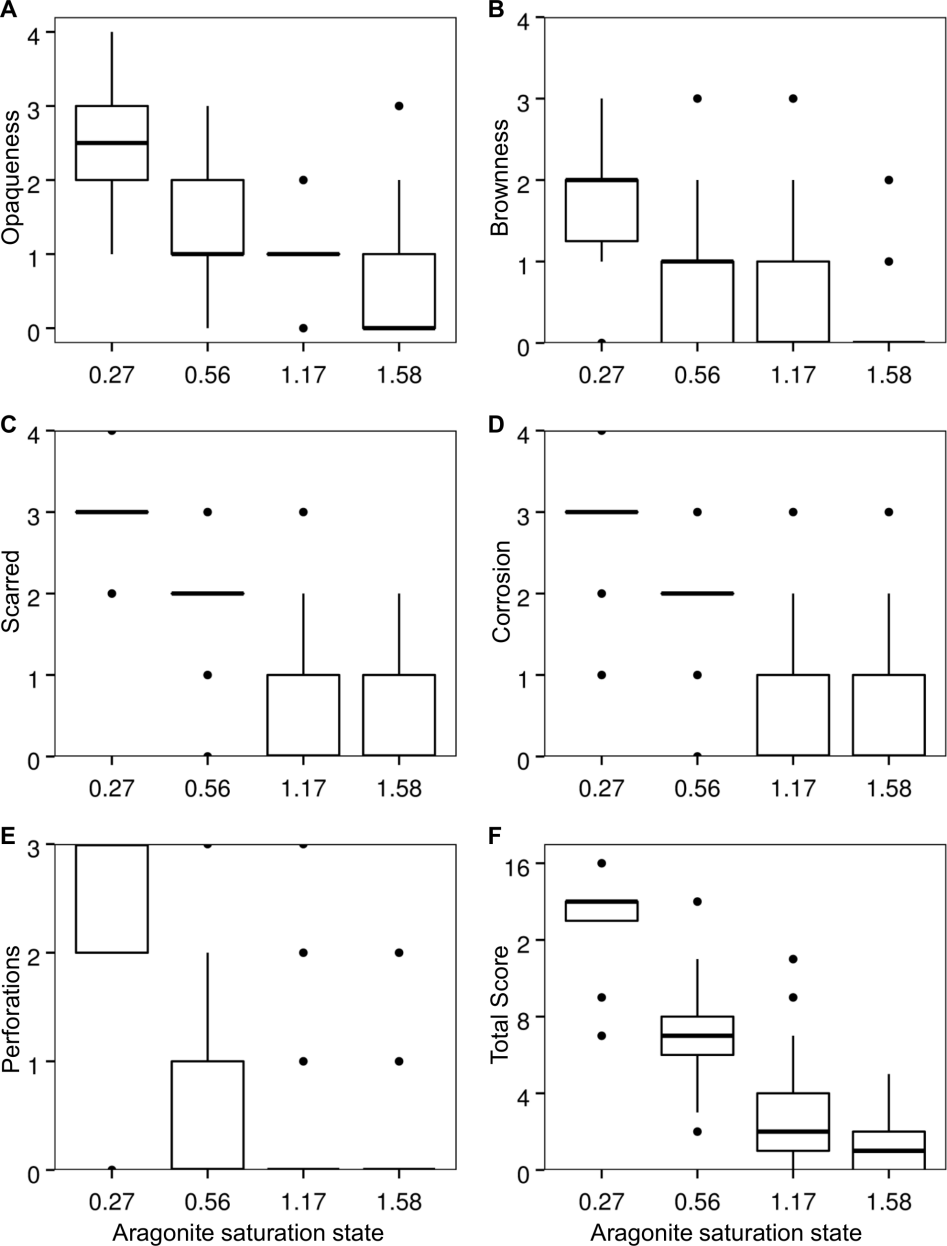
Observed pteropod shell condition scores as a function of Ω_a_ treatment. Each box plot shows the median with the upper and lower first and third quartiles. The whiskers extend to the highest and lowest values within 1.5 * the distance between the first and third quartiles. Outliers are plotted as points.

The best-fit models for shell condition and both continuous and discrete Ω_a_ data included Ω_a_ as a fixed effect and jar within experiment as a random effect (Table 2). Although residuals from the models deviate from a normal distribution (Figure 3), they show a reasonable approximation of normality such that inference is possible given the robustness of the regression approach to normality assumptions [56]. Consistent with model assumptions, a Fligner-Killeen test indicated similar variance of residuals among treatments in the discrete model (p = 0.10; Figure 4). The slope of the equation for the continuous Ω_a_ data set was negative (shell condition score = −6.08 * Ω_a_+10.09) and significantly different from zero (t = −12.56, p<0.001; Figure 5). Based on the discrete model confidence intervals, dissolution scores for the Ω_a_≈1.59 and Ω_a_≈1.17 treatments were not significantly different from each other, but scores from the Ω_a_≈0.56 and Ω_a_≈0.27 treatments differed from one another and were significantly higher than those from the oversaturated treatments. Shell condition score decreased with Ω_a_ treatment for all five shell condition characteristics (Figure 6). Pteropods preserved prior to the experiment showed little to no shell dissolution (mean shell condition score ± standard deviation = 0.5±0.6). One shell was slightly opaque (score = 1), 5 had scarred structures (score = 1 for 4 shells and 2 for 1 shell), and one had mild corrosion (score = 1).

## Discussion

**Figure 7:**
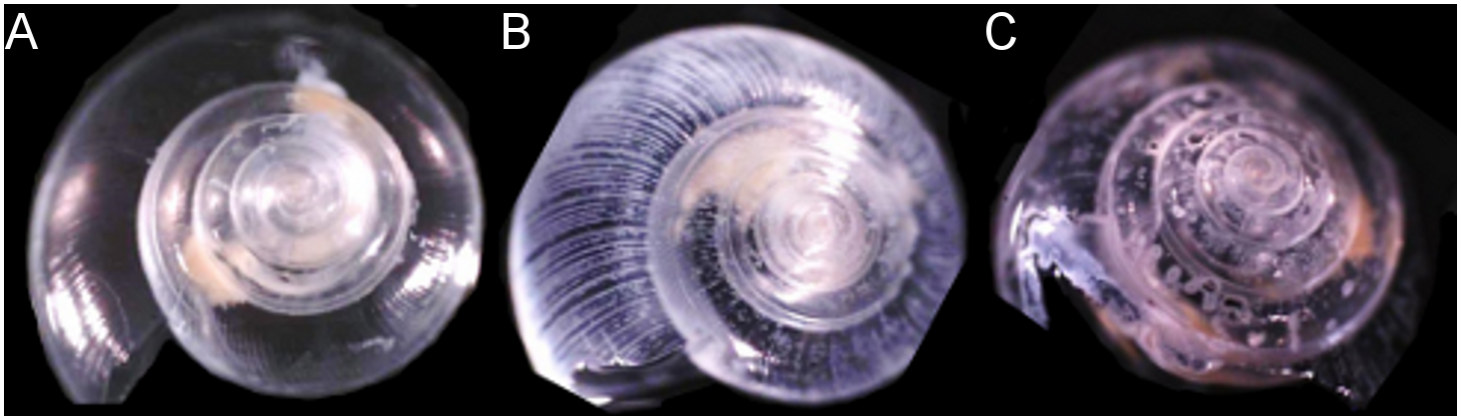
Representative shells from pteropods incubated in the (a) Ω_a_≈1.59, (b) Ω_a_≈0.56, and (c) Ω_a_≈0.28 treatments. Note corrosion on the ribs of the shell in image (b) and the shell perforations in image (c). Tissue that was not dissolved during the sodium hypochlorite incubation is visible as yellow-white material inside of the shells.

We observed significant shell dissolution in adult *L. helicina* after one week of exposure to seawater with aragonite saturation states that occur today in Puget Sound and along the US West Coast (Ω_a_≈0.56) and are expected to occur more frequently in the future (Ω_a_≈0.28; Figure 5–7) [34], [40]. The data suggest that *L. helicina* from Puget Sound, like *L. helicina* from other locales, is sensitive to aragonite saturation state conditions below 1, which supports our hypothesis that shell corrosion is linked to undersaturated conditions. Recent work has documented shell corrosion in pteropods collected along the US West Coast, likely from exposure to corrosive conditions [57]. Previous studies suggest that shells of living pteropods dissolve in slightly oversaturated conditions [24], [25], [26]; we found little to no evidence of this phenomenon in these experiments. Corrosion in the Ω_a_≈0.56 treatment was most notable on the ribs of the shell, indicating that these shell areas might be most prone to dissolution (Figure 7). We observed little to no shell dissolution in the treatment with the highest Ω_a_ (Ω_a_≈1.59), nor in shells of pteropods observed prior to the experiments.

Starvation conditions may have exacerbated shell dissolution during this and other studies on the response of pteropods to ocean acidification by reducing the energy available for calcification (*e.g.*, shell repair). Feeding during the incubations could have mitigated some or all of the influence of aragonite saturation state on shell dissolution. The influence of food availability on species response to carbon chemistry conditions has been documented multiple times in molluscs [58], [59], [60], including *L. helicina* [61], and other species, such as corals ([62], but see [63]). Exposure to laboratory starvation conditions for more than several days is known to suppress the metabolism of *L. helicina* and other pteropods [61], [64], [65], which could influence processes such as calcification. Taking a longer view, food availability throughout the season could also have influenced our results, for feeding history throughout a season influences the metabolic response of Antarctic *L. helicina* to ocean acidification conditions [61]. Unfortunately, we do not have data on primary productivity where the pteropods for this study were captured, so do not know if the study subjects were energy limited or if food conditions varied substantially among the different batches of pteropods used in this study.

While the discrete analysis found reduced survival with aragonite saturation state <0.3, the treatment condition representative of the future, it was unable to detect any effects that current carbon chemistry conditions may have on survival (broken stick model in Figure 2) [22], [23], [24]. While the latter result supports our expectation and is consistent with uncertainty in the relationship between ocean acidification and pteropod survival from prior work [22], [23], [24], the significant negative effect of the lowest saturation state treatment was counter to our expectation. No prior studies have assessed pteropod survival in response to such a low aragonite saturation state treatment.

Our ability to detect subtle impacts of carbonate chemistry on survival in current carbon chemistry conditions is limited due to the high variance in survival for a given treatment within and among experiments. Some of the variance among experiments is likely due to differing environmental conditions experienced by the pteropods prior to capture. Effects on survival at the intermediate Ω_a_ levels used in this study may be masked by the inherently sub-optimal environment of the laboratory, which may have promoted high mortality rates regardless of treatment. Specifically, in our experimental setup, pteropods suffered from a lack of food and, potentially, chemicals released by plastics in the experimental system (high mortality has been observed for newly hatched fish and crustacean larvae for some species reared in the system). Furthermore, in captivity, pteropods typically do not deploy mucus nets, which negatively influences their ability to hold position in the water column. Thus, while large effects of strongly acidified treatments on survival may be detectable in the laboratory, mesocosm studies are likely needed to more accurately test how small changes in carbon chemistry conditions affect survival in free-living pteropods. That said, shell dissolution was negatively related to Ω_a_ and, presumably, survival in the wild is related to shell condition.

The mechanism behind low survival in the highest CO_2_/lowest aragonite saturation state treatment is unclear. Mortality in this treatment could be related to mechanical and/or energetic complications caused by severe shell corrosion or due to other physiological effects of high CO_2_ conditions, such as those caused by regulation of acid-base balance. High CO_2_ conditions can impair acid-base balance in molluscs, in some cases leading to internal shell dissolution while compensating for extracellular acidosis [66]. This phenomenon could have caused the mean shell dissolution score for the highest CO_2_ treatment to be higher than what was predicted by the best-fit linear model (Figure 3, compare open diamond to solid line). Furthermore, in some pilot work using the same experimental setup described here (July 2012), we found that the shell-less, gymnosome pteropod *Clione limacina* also experienced low survival in the highest CO_2_ treatment compared to the lowest CO_2_ treatment, suggesting that pteropod mortality in this treatment may be due to processes unrelated to the shell (highest CO_2_ treatment: 0 of 12 alive at day 3 of incubation; lowest CO_2_ treatment: 11 of 12 alive at day 3 of incubation, 11 alive at day 9, 2 alive at day 15).

Given the sensitivity of north Pacific *L. helicina* shell condition to current carbon chemistry conditions demonstrated by this study and observations from the field [57], the microhabitat in which *L. helicina* lives and its natural history may be important for the persistence of the species in this region. Shell dissolution in *L. helicina* from the US West Coast and Southern Ocean is related to the prevalence of undersaturated conditions, indicating that some *L. helicina* populations are already affected by ocean carbon chemistry conditions, though potentially in a patchy manner [26], [57]. Little is known about the basic natural history of the species in Puget Sound, including its seasonal and spatial distribution and whether populations persist in the estuary year-round. Puget Sound populations of some zooplankton species (e.g., *Calanus mashallae*) appear to be largely re-initiated every season via transport from the Pacific Ocean (Frost and Pierson, unpublished data). Pteropods may follow a similar mechanism, flushing into Puget Sound every spring following the increased estuary circulation produced by spring freshwater run off [67]. A highly relevant question is whether the pervasive extent of waters undersaturated with respect to aragonite in the winter months makes Puget Sound a population sink that requires reseeding from the coast for persistence. Research on overwintering, juvenile *L. helicina* from other regions finds shell dissolution at carbon chemistry conditions currently found in Puget Sound in the winter [23], [24]. Laboratory research on whether calcification can balance dissolution in the variable conditions found in Puget Sound could inform research on this hypothesis [22].

Northeast Pacific *L. helicina* are typically found above 100 m [27], which is shallower than in other ocean regions [29]. Investigations to explore whether this is a behavioral modification to avoid low pH, corrosive waters could be a fruitful line of research for understanding how current variation in and future changes of carbon chemistry condition may influence free-living populations. Behavioral research could also explore whether pteropods in Puget Sound and elsewhere seek out energy-rich phytoplankton blooms, a strategy that would simultaneously lead them to low CO_2_ waters. Unlike Bednaršek et al. (2014), we observed little to no corrosion in pteropod shells preserved soon after collection. This finding suggests that the carbon chemistry conditions experienced by these pteropods were non-corrosive, pteropods with shell corrosion repaired their shells, or pteropods with corroded shells do not persist long in free-living populations.

We found that, under starvation conditions in the laboratory, pteropods collected from Puget Sound are sensitive to current and future local carbon chemistry conditions. We note that these laboratory data are insufficient to even conjecture about the trajectory of regional pteropod populations because of the potential influence of starvation conditions on response to our treatments and other influences on pteropod population dynamics. For example, changes in ocean chemistry projected to occur due to climate change could impact pteropod survival in Puget Sound. Low salinity events, which will be more frequent in the estuary under the projections of higher winter rainfall [68], may exacerbate the impacts of ocean acidification on wintertime mortality and shell dissolution, as they do in a closely related species, *L. retroversa* [69]. Furthermore, laboratory experiments on other species that are highly sensitive to acidification do show variation in the response of individuals to low pH conditions, indicating that the potential for evolution in response to ocean acidification should not be ignored [43], [44], [70].
